# Multi-functional approach in the design of smart surfaces to mitigate bacterial infections: a review

**DOI:** 10.3389/fcimb.2023.1139026

**Published:** 2023-05-23

**Authors:** Shobana Rajaramon, Helma David, Anusree Sajeevan, Karthi Shanmugam, Hrithiha Sriramulu, Rambabu Dandela, Adline Princy Solomon

**Affiliations:** ^1^ Quorum Sensing Laboratory, Centre for Research in Infectious Diseases (CRID), School of Chemical and Biotechnology, SASTRA Deemed to be University, Thanjavur, India; ^2^ Department of Industrial and Engineering Chemistry, Institute of Chemical Technology, Bhubaneswar, India

**Keywords:** bio-inspired, antibacterial biomaterials, anti-biofilm, antimicrobial therapies, micropatterning, biomaterial, anti-adhesive

## Abstract

Advancements in biomedical devices are ingenious and indispensable in health care to save millions of lives. However, microbial contamination paves the way for biofilm colonisation on medical devices leading to device-associated infections with high morbidity and mortality. The biofilms elude antibiotics facilitating antimicrobial resistance (AMR) and the persistence of infections. This review explores nature-inspired concepts and multi-functional approaches for tuning in next-generation devices with antibacterial surfaces to mitigate resistant bacterial infections. Direct implementation of natural inspirations, like nanostructures on insect wings, shark skin, and lotus leaves, has proved promising in developing antibacterial, antiadhesive, and self-cleaning surfaces, including impressive SLIPS with broad-spectrum antibacterial properties. Effective antimicrobial touch surfaces, photocatalytic coatings on medical devices, and conventional self-polishing coatings are also reviewed to develop multi-functional antibacterial surfaces to mitigate healthcare-associated infections (HAIs).

## 1 Medical devices –a major concern in HAI?

Advancements in biomedical technology have equipped the healthcare sector with a breathtaking array of medical devices, including implantables, to ameliorate prevention, diagnosis, treatment, and prognosis and alleviate medical conditions to improve the quality of life. A significant impediment to the effective functioning of medical devices emanates from microbial contamination leading to the development of infections contributing to HAI. The hike in medical device utilisation has been reflected in increased incidences of HAI. HAI progress report 2019, published by the Centres for Disease Control and Prevention (CDC), cites 72,000 deaths among hospitalised patients (CDC, 2020), and 50 to 70% of healthcare-associated infections are attributed to medical device-related infections (Bryers, 2008). The frequency of device-related infections is underestimated owing to the need for advanced non-invasive diagnostic methods to assess microbes on implanted devices, non-culturable microbes, and small colony variants that cannot be detected with traditional culture-based methods (Percival et al., 2015). The rates of infection of most common medical devices have been reported elsewhere (VanEpps and Younger, 2016). The device-linked mortality rate varies from <5% as with dental implants to >25% observed with mechanical heart valves (Darouiche, 2001).

According to WHO, the incidence of HAIs escalated 19 times in developing countries compared to reported cases in Germany and the USA, with the employment of invasive, indwelling devices, especially central line catheters, urinary catheters, heart valves, and ventilators, due to the risk of exposure to pathogens (WHO fact sheet). Using catheters is inevitable in most procedures to deliver fluids, food, drugs, body fluid collection for diagnosis, and ECMO. This resulted in central line-associated bloodstream infections (CLABSI), including sepsis and catheter-associated urinary tract infections (CAUTI), major HAIs (Haque et al., 2018). Patients implanted with cardiovascular implantable devices such as heart valves, pacemakers, and implantable automated defibrillators pose an increased risk for endocarditis and high mortality rates(Greenspon et al., 2011; Bradshaw et al., 2014). The cases of calcification of silicone implants used in breast reconstruction and failure of orthopedic implants can be due to undiagnosed microbial contamination. Implantable device-related infections can be devastating as the first-line treatment involves the usage of broad-spectrum antibiotics, and inappropriate use can result in systemic toxicity and the emergence of antimicrobial resistance. The ultimate effective course of action resorts to surgical debridement, implant retrieval, and a partial or total revision of the medical procedure (Francolini et al., 2020). Microbial contamination of sutures, which is common in surgical procedures, can lead to chronic infections and delay wound healing. Moreover, wound dressings and sutures have been paralleled with bioreactors for the proliferation and growth of microbes (Percival et al., 2015). In the current COVID times, viral contamination of protective devices, including masks, gloves, face shields, and protective suits, also increases the risk of contracting the infection (Tang et al., 2020).

External environmental sources can contaminate devices through inhalation; ingestion breaks in the skin barrier and mucous membranes following surgical intervention, endogenous sources of opportunistic pathogens in microflora, and hematogenous circulated pathogens contacting the device surface (von Eiff et al., 2005; Arciola et al., 2018). The CDC has recommended guidelines for clinical interventions to reduce device-related infection rates, including but not limited to the appropriate selection of patients for catheterisation, the reasonable duration for catheterisation, hygiene, and aseptic practices. These measures alone cannot be sustainable for combatting device-related healthcare-associated infections. Their infection-limiting effects can wane with increasing hospitalised patients and the emergence of antimicrobial resistance. The CDC report 2019 also mentions the annual occurrence of antibiotic-resistant infections of over 2.8 million in the US and 35,000 deaths due to treatment futility. By 2050, the current rise in antimicrobial resistance rate can cause 10 million deaths yearly (O’Neill, 2016). Over the years, endemic device-related healthcare-associated infections have been a matter of great concern due to increased re-hospitalization rates which in turn increase the risk of contracting infections, high morbidity and mortality rates, the emergence of AMR, additional costs for the healthcare system and hospital resources, and high healthcare expenditures for patients augmenting the burden of healthcare instead of reducing the burden of illness (Allegranzi et al., 2011). Thus, the global market necessitates looking into the cause and designing innovative solutions to control HAIs.

## 2 Bacterial biofilms – an emerging cause of HAIs?

The formation of biofilms on medical devices was first recognised in 1972 as a cause of device-related infections specifically associated with ubiquitous biofilms on catheters and cardiac pacemakers (Johanson, 1972; Francolini et al., 2020). Bacterial biofilms are ascribed to 65% - 70% of bacterial infections related to medical devices (Olmo et al., 2020). Biofilm formation on medical equipment, surgical tools, and implantables, including biosensors, medical clothing, and water purification systems, poses the threat of acquiring HAI. The major bacterial species isolated from clinically retrieved implants comprise gram-positive bacteria, including *Enterococcus faecalis*, *Staphylococcus aureus, Staphylococcus epidermidis*, and *Streptococcus viridans*; and gram-negative bacteria - *Escherichia coli, Klebsiella pneumoniae, Proteus mirabilis*, and *Pseudomonas aeruginosa*(Donlan, 2001). In particular, *S.aureus* and *S.epidermidis* interspecies biofilms source about 50% of medical device-related infections (Nowakowska et al., 2014). The leading HAIs are predominantly associated with biofilm-forming multidrug-resistant strains of methicillin-resistant *Staphylococcus aureus* (MRSA), vancomycin-resistant *Enterococci* (VRE), *Clostridium difficile*, coagulase-negative *Staphylococci*, and multi-drug resistant gram-negative bacilli (Al-Tawfiq and Tambyah, 2014). Medical devices are more vulnerable to bacterial colonisations, even in the presence of less bacterial load, owing to the physio-chemical properties of the foreign body (implanted device) and lack of vascularisation compared to host tissues (Vergidis and Patel, 2012; Khatoon et al., 2018). Biofilms are rapidly formed on endotracheal tubes, and pathogens are correlated to the microflora of the lower respiratory tract (Adair et al., 1999). Bacterial colonisation on indwelling devices such as dental implants can disperse and lodge at another niche within the body to form biofilms, which may result in HAI-like infective endocarditis, cystic fibrosis, bacteremia, and chronic wounds.

The medical devices within the body are rapidly coated with proteins, including fibronectin, vitronectin, fibrinogen, collagen, thrombospondin, laminin, and polysaccharides from ECM, blood, interstitial fluid, and immune components (Franz et al., 2011). The protein adsorption on implants, associated with the Vroman effect in some cases, is pivotal in bacterial adherence and initiation of biofilm formation, which is essential in the pathogenesis of device-related infections. Bacteria initially adhere reversibly to the protein-conditioned or unconditioned medical device surface via nonspecific forces, including hydrophobic interactions, steric forces, electrostatic interactions, Van der Waals forces, and acid-base interaction forces (Hori and Matsumoto, 2010; Mao and Fang, 2020). The exchange of bacteria with the device surface is influenced by the surface properties of bacteria and medical devices, conditioning proteins, and the composition of the surrounding medium, including ionic strength (Khalid et al., 2020). Thermodynamically, bacteria with hydrophobic membranes prefer hydrophobic device surfaces for attachment. The interactions of bacterial cell wall components and surface structures such as adhesins, including pili, flagella, proteins, and lipopolysaccharide chains and autolysins with collagen, fibronectins, and fibrinogen in conditioned film led to a strong irreversible attachment (Speziale et al., 2009; Hori and Matsumoto, 2010). For instance, an adhesin that is a covalently anchored cell wall protein (SasX) facilitates adhesion, plays a vital role in the virulence of *S.aureus*, and is associated with the growth of MRSA (William da Fonseca Batistão et al., 2016). *S.aureus* produces multifunctional effectors - cell-wall anchored microbial surface components recognising adhesive matrix molecules (MSCRAMMs) that facilitate adhesion to biomaterial surface conditioned by serum proteins fibrinogen, collagen, and fibronectin and may also attenuate host immune response in favour of biofilm formation (Speziale et al., 2009; VanEpps and Younger, 2016). Irreversible attachment of pathogens mediated by autolysin, AtlA, undergo proteolytic cleavage to produce amidase that binds to matrix proteins fibrinogen, fibronectin, and vitronectin, thereby allowing *S.aureus* to attach to conditioned abiotic surfaces. *S.epidermidis* adhere to polymeric devices by surface-associated autolysin (AtlE) and other medical devices through teichoic acids by binding to adsorbed fibronectin (Arciola et al., 2018).

In the transition from planktonic to the sessile state upon irreversible attachment, bacteria divide, proliferate, and produce a slimy extra polymeric substance (EPS), which protects the sessile bacteria from host immune response and antibiotics. The EPS production by pathogenic bacteria is upregulated when there is differential gene expression and phenotypic shift due to the presence of quorum sensing molecules, teichoic acids, proteases, nucleases, and phenol soluble modulins. Subsequently, microcolonies are formed with bacteria embedded in EPS. The released EPS activates cyclic dimeric guanosine monophosphate (C-di-GMP), an intercellular signaling molecule, to stimulate bacterial species’ proliferation and strong attachment. Subsequently, the synthesised exopolysaccharide, pentasaccharide, glucose-rich polysaccharide, and alginate signal produce more C-di-GMP leading to thicker and stronger biofilms (Khatoon et al., 2018). New microbes are also recruited by embedded bacteria via chemo-attractants and signalling mechanisms (quorum sensing) and form mature biofilms. Extracellular DNA in EPS also improves the strength and stability of the biofilm matrix by modulating the innate immune response (Thurlow et al., 2011). The adherence between *S.epidermidis* and *S.aureus* within a multispecies biofilm is enhanced by polysaccharide intercellular adhesin (PIA), a biofilm matrix component that increases interconnections within the matrix. PIA synthesis is upregulated under stress conditions in *S.epidermidis*, resulting in higher resistance to aminoglycoside antibiotics (Arciola et al., 2005). Extracellular DNA and resistance plasmids in the biofilm matrix may be transferred to adjacent bacterial cells to become resistant to antimicrobials (Roberts and Kreth, 2014). Thus, the biofilm formation on medical devices contributes to HAIs that are resistant to antimicrobial treatment and leads to the persistence of infections. This provides a future outlook on designing smart medical surfaces to hinder biofilms and potentiate the action of antimicrobials.

## 3 Integrative design of smart medical device surfaces: hindering the bacterial biofilms?

The tenacious appendage of bacteria to the device surface is the root cause of biofilm development leading to infections. The intended properties of the medical devices can be altered by fouling. However, prevention of the formation of biofilms is possible and considered superior to mitigate infections rather than treating mature biofilms as they are more tolerant to stress conditions (Subhadra et al., 2018). The biofilm prevention strategy improves the success of the intended function of medical devices and prolongs the life of the device. The next-generation biomaterials with anti-infective properties are the need of the hour to provide a sustainable solution to mitigate the challenges of existing release-based chemical modifications of the surface and AMR) (**Table 1**).

**Table 1:** Topography modifications and their biological efficacy to control growth/biofilm on surfaces.

Surface	Inspiration & topography	Surface considered	Tested pathogens	Outcomes	Inference	References
Anti-bacterial	*Psaltoda claripennis wings-* nanostructured surface	*Magicicada ssp.*,(Brd II) *Tibicen* ssp., (DD), *Pogomphus obscurus* spp (DF) wings	*1. S.cerevasiae*	1. Reduced viability 2. Loss of membrane integrity	1. Greater cell rupturing in higher aspect ratio nanoscale features (DD & DF)	(Pogodin et al., 2013; Nowlin et al., 2015)
	*Psaltoda claripennis* wings- with longer & shaper nanopillar topography	*-*	*1. P.aeruginosa* *2. S.aureus*	1. Killed 95% of *P.aeruginosa &* 83% of *S.aureus*	1. Bactericidal efficiency higher than normal pillar topography due to high mechanical energy	(Ivanova et al., 2020)
	Nanopillar topography, with random spacing	Titanium black metal surface	1. *E. coli* 2. *P. aeruginosa*, *3. M. smegmati* *4. S. aureus*	1. Killed all tested pathogens *(<* 4h, 90% - 98%) except *S.aureus* 2. Less effect on *S. aureus (22% -* 4* h* & 76% -24 h) 3. Proliferation of hMSCs	1. High efficiency due to the different geometry of the nano architecture when compared to the cicada wing surface	(Hasan et al., 2017)
	Dragonflies & cicada wings - nanopillar topography	Titanium dioxide (TiO_2_)	1. *E. coli* 2. *K.pneumoniae* 3. *S. aureus*	1. Induced oxidative stress response 2. *E. coli* & *K. pneumoniae* (1000 fold reduction- < 6h) compared to *S.aureus*	1. Penetrate into *S. aureus at* a lower frequency due to high turgor pressure & rigidity	(Jenkins et al., 2020)
	Nanoknives or nano blades	Graphene sheets	1. *E.coli* *2. S.aureus*	*1. E.coli* less susceptible compared to *S.aureus*	1. Due to the extra outer membrane in gram-positive bacteria	(Akhavan and Ghaderi, 2010)
Anti-adhesive	Sharkskin - Sharklet micropatterned topography	poly(dimethyl siloxane) elastomer (PDMSe)	*1. S.aureus*	1. Sharklet AF™ prevented early biofilm colonisation (>21 days)	–	(Chung et al., 2007; Graham and Cady, 2014)
	Sharklet micropattern	–	*1. S.aureus* *2. P.aeruginosa*	1. Adherence was reduced (92.3 -99%) 2. Restricted transference (>90%)	–	(Xu et al., 2017)
Super-hydrophobic	Lotus leaf- air entrapment between the Micro/ nanostructures	TiO_2_ nanotubes	*1. S.aureus* *2. E.coli*	1. Prevents bacterial adherence & biofilm	–	(Patil et al., 2018)
		1H,1H,2H,2H-perfluorooctyltriethoxysilane, P25 TiO_2_ nanoparticles	1. *S.aureus* 2. *E.coli* 3. *MRSA* 4742	1. Prevents bacterial attachment (<4h) 2. After 24 h 93–99% adherence	1. Loss of air-bubble interface, less superhydrophobicity	(Hwang et al., 2018)
	*Cicada wings*	–	*1. B*. *subtilis* *2. B*. *catarrhalis* *3. E*. *coli* *4. P*. *maritimus* *5. P*. *aeruginosa* *6. P*. *fluorescens* *7. S*. *aureus*	*1. *Irregular morphology in gram-negative bacteria exhibiting lethal conditions. *2. *Morphologies remained unchanged in gram-positive	1. Thick peptidoglycan layer provides rigidity to gram-positive bacteria	(Hasan et al., 2013)
Slippery liquid-infused porous surface (SLIPS-omniphobic)	*Nepenthes* pitcher plant - Thin lubricating film coating	Polyfluoroalkyl- silanised enamel surface was infused with Fluorinert FC-70 lubricant	*1. S.mutans*	1. Sparse and isolated bacteria growth (24h) 2. Minimal growth by 48h. 3. Less dental plaque in SLIPS incisors	1. Overcome the drawback of the superhydrophobic layer. 2. Lubricating thin film coating for the liquid droplets to slide away.	(Yin et al., 2016)
	–	Polycarbonate, polysulfone and polyvinyl chloride (PVC) tethered with liquid perfluorocarbon surface (TLP)	*1. E. coli* *2. P. aeruginosa*	1. Suppressed biofouling & biofilm formation	–	(Leslie et al., 2014; Abdulkareem et al., 2022)
Photocatalytic	–	Glass surfaces and glass microfibre filters coated with crystalline nanostructured TiO_2_	*1. S.aureus* *2. P.putida*	*1. *After 2 h of visible/near UV light irradiation cells *2. *Membrane damage.	1. Membrane damage due to ROS, intermediates of oxygen-dependent photosensitised reactions.	(Jalvo et al., 2017)
	–	Phosphorus (P)- Fluorine (F) modified TiO_2_	*1. E.coli* *2. S. epidermidis* *3. P. fluorescens*	1. Reduced colonisation (99%)	–	(Yan et al., 2020)
	–	copper (Cu)-doped TiO_2_ (Cu-TiO_2_)	*1. E.coli* *2. S.aureus*	1. No significant change in the dark. 2. Bacterial reduction under visible light irradiation (5-Log reduction)	–	(Mathew et al., 2018)
Self-polishing	Prevention of biofouling on the marine hull	Alternative layer-by-layer (LbL) deposition of dextran aldehyde (Dex-CHO) and carboxymethyl chitosan (CMCS) on Stainless steel	*1. E.coli* *2. S.aureus*, *3. Amphora coffeaeformis*	1. Attachment & lethality were directly proportional to the number of assembled bilayers	1. Increase in Dex-CHO/CMCS bilayers is directly proportional to surface hydrophilicity 2. Decrease in surface roughness, antimicrobial & antifouling surface	(Xu et al., 2018)

### 3.1 Bioinspired nanostructured medical device surface

Nature, a source of inspiration to engineers and researchers, provides solutions for problems in various fields through the appreciation of intriguing sophistication and miniaturisation that has evolved through many years. The biomimetic strategy involves replicating the surface topography, morphological features, and chemical concepts from nature to change the surface functionality and improve the ability of the surface to kill or repel bacteria. The medical device surfaces are manipulated to possess multifunctionality to decimate bacteria that contact the surface (anti-bacterial) and prevent the adhesion of bacteria to the surface (anti-adhesive). In this milieu, surface roughness in terms of nano-topography is the most critical parameter to attain antifouling or bactericidal properties, and surface nanoroughness ranging from 30 nm to 1 μm efficiently reduces the attachment of bacteria (Medilanski et al., 2002; Fröjd et al., 2011; Bazaka et al., 2015). Various nano or micro topographical modifications for next-generation medical device surfaces have been explored, including micro/nanopores, micro ridges, micro/nanopillars, nanocolumns, nanocolumns, nanowires, nanorings, nanospinules or hairs, and nano spikes/needles (Reddy et al., 2011; Tripathy et al., 2017; Yang et al., 2022). The interaction between the substrate topographies and bacteria leads to bacterial killing or preventing bacterial adherence and circumvents biofilm formation, mitigating AMR (Feng et al., 2015; Khalid et al., 2020).

#### 3.1.1 Anti-bacterial surface

**Figure 1:**
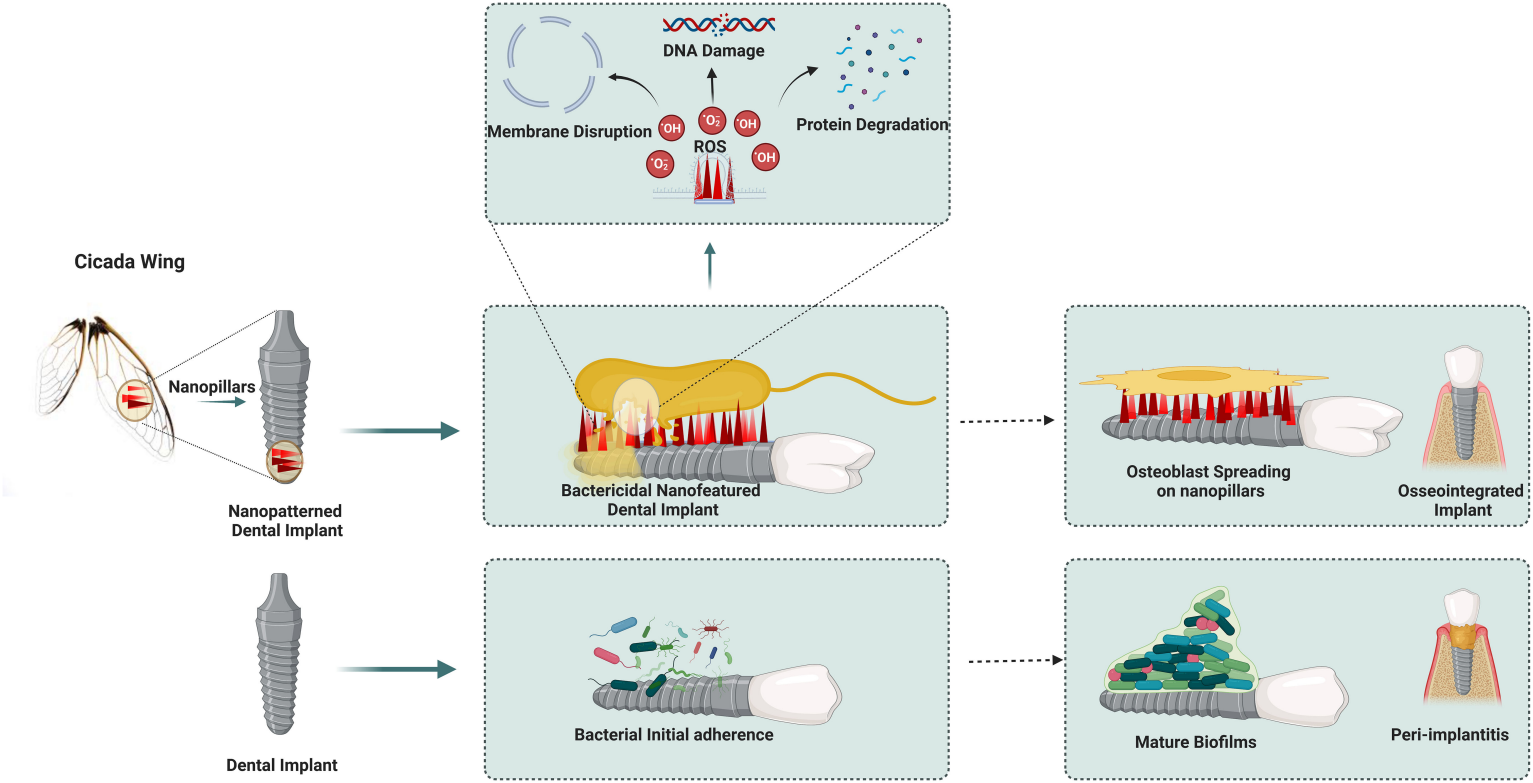
Cicada nanopillar topography augments the bactericidal action via., membrane stretching.

**Figure 2:**
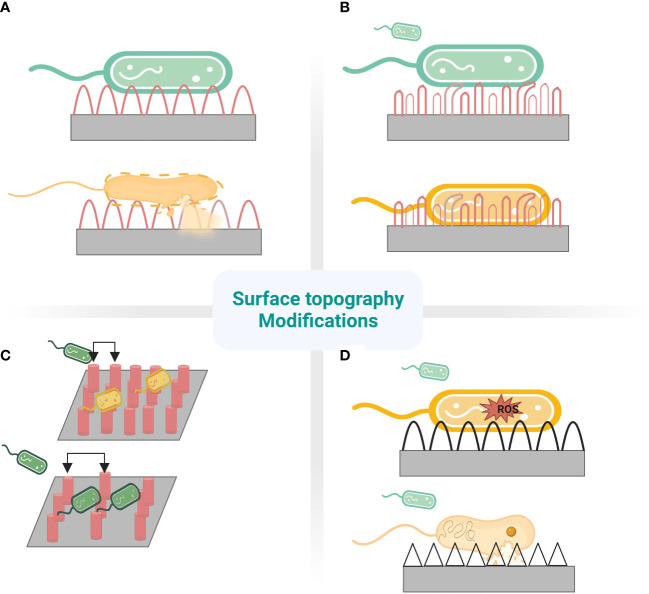
Nanostructures mechanism to prevent fouling. **(A)** cicada wing- Stretching **(B)** dragonfly wing- tearing **(C)** different pitches of pillars.Top:480nm-bactericidal. Bottom:1100nm-bacteria pattering **(D)**Top: ROS mediated. Bottom: Penetration.

Bactericidal surfaces with nanostructures annihilate bacteria on interaction with the surface by exerting mechanical forces (Patil et al., 2021). Interestingly, the wings of some insects like cicada and dragonflies possess bactericidal activity naturally, which is attributed to the nanopillar pattern on their wings (**Figure 1**)(Hasan et al., 2019; Larrañaga-Altuna et al., 2021). The cicada (*Psaltoda claripennis*) insect wings inspired nanopillar topography to induce stretching of the adsorbed bacteria along nanopillars, leading to rupture of the bacterial cell membrane due to lower elasticity of bacterial membrane than adhesion energy (Kelleher et al., 2016). The pioneering implementation of cicada wing-inspired nanopillars of 200nm height, 100nm base diameter tapering to 60nm at the tip with the inter-structure distance of 170nm could lyse the gram-negative bacteria *P.aeruginosa* at a rate of 2.05×10^5^ min^-1^cm^-2^ colony forming units as explained by the biophysical model (**Figure 2A**) (Ivanova et al., 2012; Pogodin et al., 2013). However, the mechanism was ineffective on gram-positive bacteria owing to the significant rigid cell wall due to a thick peptidoglycan layer (Hasan et al., 2013). Eukaryotic *Saccharomyces cerevisiae* was also susceptible to rupture, similar to bacteria, implying the broad-spectrum antimicrobial activity of cicada nanopillars. This mechano-bactericidal mechanism to rupture the cells is highly dependent on the adhesion ability of microbes to the surface and independent of the surface composition when experimented with the gold-sputtered surface (Nowlin et al., 2015). Bactericidal activity has been enhanced with high aspect ratio nanostructures like flexible silicon nanopillars due to auxiliary lateral stretching of the bacterial cell membrane depending on the interaction of nanopillars at the cell edge and the height of nanostructure attached to the bacterial cell membrane. The sharper and longer nanopillars, with heights ranging from 220-360 nm, produce higher bactericidal efficiency (killed 95% *P.aeruginosa* and 83% *S.aureus*) as it stores more considerable mechanical energy (i.e. elastic energy) on deformation, which translated into the pressure applied on the bacterial cell membrane bacterial mobility effectuating lethal shear forces upon creeping on unfavourable topography (Ivanova et al., 2020; Mao and Fang, 2020). The effect of nanopillars on *E. coli* and *S. aureus* differed as *E.coli* cell divides by elongation resulting in lethality induced by nanostructures to daughter cells. In contrast, *S. aureus* daughter cells cluster on the original cell and dodge the bactericidal effects of nanostructures (Lin et al., 2018). In experimental studies to develop a nanopillar surface of a medical device with improved broad-spectrum bactericidal effect, the metal-organic framework (MOF) was positively charged to attract negatively charged bacterial cell walls to its surface and rupture them (Riduan and Zhang, 2021). However, the killing rate depends on the bacterial species and surface nanostructures under consideration. For *P.aeruginosa*, time-lapsed AFM and CLSM studies have confirmed that cicada nanopillars and their silicon replicas kill their adsorbed bacteria within ~5-10 minutes and release the debris within ~20 minutes of cell rupture to preserve its bactericidal properties (Ivanova et al., 2012; Nguyen et al., 2019). Surface topographies should be optimised for the intended bactericidal application. Generally, nanostructures must be sufficiently dense to prohibit bacteria from escaping by contacting cavities between nanostructures, experiencing less or no suspension force. The bactericidal activity of nanopillar revolves around the height, sharpness, width, and spacing between the array of pillars (Kelleher et al., 2016). Spacing is essential to avoid the mere resting of bacteria on nanostructures without suspending the bacterial membrane on the nanostructure array. There are no standard topographical parameters for established comprehensive bactericidal activity. However, most inspected bactericidal parameters range from 100-1000nm in height, 10-300nm tip diameter, and interspacing distance of less than 500nm (Modaresifar et al., 2019).

Bactericidal nano-featured surfaces have been developed on various substrates, including Titanium (Ti), Silicon (Si), Aluminium (Al), glass, and polymers, in support of the biophysical model. Anisotropic randomly oriented nanopillar surface was fabricated on Ti by etching process exhibited broad-spectrum bactericidal property and cytocompatibility. The etched titanium appears black owing to the presence of nanostructures with dimensions of 1µm height and an average diameter of 80 nm. This multi-biofunctional Ti surface ruptured gram-positive and gram-negative bacteria, including *E. coli, P. aeruginosa, S. aureus*, and *M. smegmatis* with multi-fold efficiency compared to polished Ti surface. Moreover, it enhances the attachment and proliferation of human mesenchymal stem cells (hMSCs) and encourages differentiation to osteogenic lineage in the presence of relevant factors *in vitro* (Hasan et al., 2017). Bacterial cells being rigid compared to mammalian cells are lysed by nanostructures, while elastic mammalian cells adhere with the help of integrins and spread over the nanostructures, recognising them as anchorage points and proliferating. Thus, an optimally modified implant surface allows tissue cells to win the race for the surface and effective implant integration without developing bacterial infections (Modaresifar et al., 2019; Ishak et al., 2020). Microfluidic experiments demonstrated black silicon nano spiked substrate to be functional under fluid flow. Thus, nanostructured surfaces can be employed in the inner surface of urinary catheters (Wang et al., 2016). In addition to the biophysical model involving the rupture of bacterial cells, it has been proposed that Titanium dioxide (TiO_2_) nanopillars impede bacterial cell division and proliferation and induce reactive oxygen species(ROS) production. Biomimetic TiO_2_ nanopillars penetrated and deformed the bacterial membrane, altering the genetic expression in response to mechanical stress (**Figure 2D**). The lack of expression of fimbria appendages by *E. coli* and *K. pneumoniae* evidences this. ROS production within bacterial cell increase differential expression of oxidative stress and repair proteins such as superoxide dismutase and methionine sulfoxide reductase in *S. aureus*. The generated ROS increased the susceptibility of membrane and cellular components to damage, culminating in the degradation and lysis of bacterial cells (Jenkins et al., 2020). There is no consensus on a model explaining the comprehensive bactericidal effect of nanostructured surfaces. It is also challenging to arrive at, owing to the complex interaction between viscoelastic bacterial membranes with appendages and surface nanostructures inspired by various biological examples. Further, the interaction is influenced by the *in vivo* local factors. These also make it challenging to attribute specific interaction forces requisite for a bactericidal effect.

Graphene and its derivatives as 2D nanomaterials have been extensively studied for broad-spectrum antimicrobial properties contributed by their multifunctional properties: increased stability and surface area, high biocompatibility, and uncomplicated surface modification (Pandit et al., 2021). Graphene nanosheets, regarded as nanoknives or nano blades, are aligned vertically on the device’s surface as the orientation angle strongly influences antimicrobial activity. The strong interaction between the phospholipid’s lipid bilayer and lipophilic graphene causes the puncturing of bacterial membranes or the formation of pores, leading to mechanical cell disruption (Tu et al., 2013; Radhi et al., 2021). Additionally, ROS generation and disturbance in the redox reaction by graphene affects the cellular metabolism, which, together with the other effects, results in broad-spectrum bacterial inactivation (Krishnamoorthy et al., 2012). The loss of membrane potential due to the conductive nature of graphene and ATP depletion due to interruption in the electron transport chain leads to cell death (Syama and Mohanan, 2019; Mohammed et al., 2020). In accordance with this mechanism, graphene sheets were used as nano blades against *E.coli* and *S. aureus*. *S. aureus* was more susceptible to killing than *E.coli* due to the extra outer membrane in gram-positive bacteria, although the peptidoglycan layer is thinner than in gram-negative bacteria (Akhavan and Ghaderi, 2010). The sharp monolayered edges and increased lateral area of nano blades are known to boost the bactericidal activity of the nano knife by allowing for the extraction of large patches of membrane phospholipids (Mohammed et al., 2020). Dense, sharp functionalised graphene sheets with 10-15 nm edge provided heightened broad-spectrum antibacterial activity under membrane pore formation, altering the bacterial cell’s osmotic pressure, causing membrane potential loss, and leakage of cytoplasmic materials leading to lysis (Chen et al., 2014). For graphene nanostructures, biocompatibility has been reported upon functionalisation with polyethylene glycol, polyethyleneimine, and bovine serum albumin, but contradictory cytotoxicity dependent on concentration, size, and shape are also reported (Linklater et al., 2018). Graphene nanostructures less than 5nm may get inserted into the mammalian membrane and subsequently internalised by macrophages, while mammalian cells may spread and wrap around larger graphene nanostructures. Hence, biocompatibility and cytotoxicity must be assessed before implementation (Lin et al., 2018).

The durability of bactericidal nanostructures is inconclusive due to the need for long-term experiments in various *in vivo* conditions. The possibility of nanostructures fragmenting from the device surface, exceptionally flexible nanostructures with weak modulus, raises the concern of loss of antibacterial activity over time *in vivo* and cytotoxicity to the mammalian cells (Lin et al., 2018; Ishak et al., 2020). The robustness of the bactericidal effect of the nanostructured surface following inevitable protein conditioning on implantable devices is also still being determined. Nanostructured surfaces encountered by high bacterial load may be contaminated by bacterial debris, leading to inflammation due to immune responses. Nanostructured surfaces kill encountering bacteria and potentially prevent biofilm formation, but it jeopardises host microbiota. So, the possibility of manipulating surface chemistry through the functionalisation of the nanostructures to increase the lysing rate of pathogenic bacteria and the specificity of the bactericidal action towards certain pathogenic bacterial species can be considered in the design of the device surface (**Figure 2**).

#### 3.1.2 Anti-adhesive surfaces

**Figure 3:**
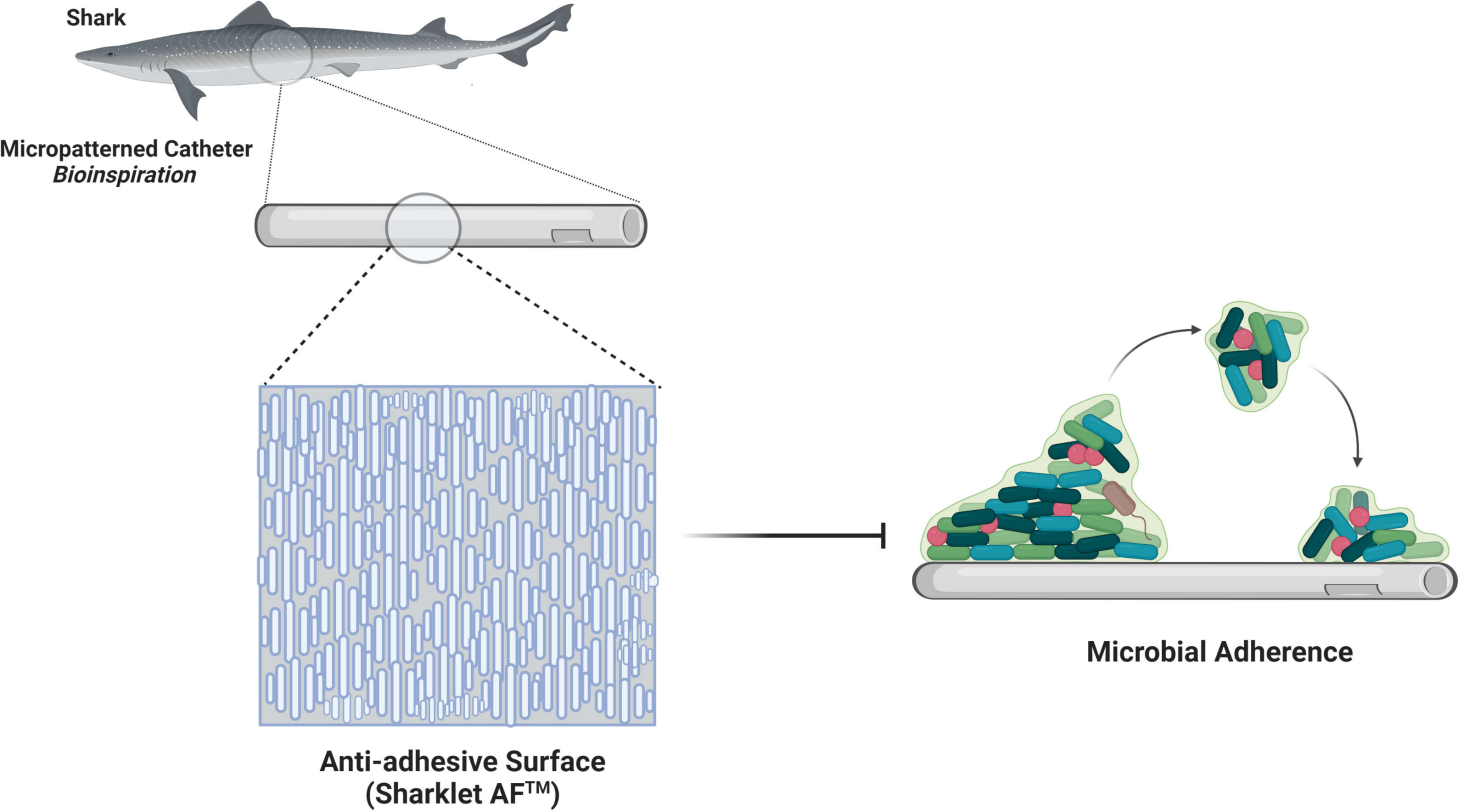
Sharkskin-inspired sharklet micropatterned surface modification (Sharklet AF™) designed to prevent bacterial adhesion. This property is contributed by a series of diamond-shaped assemblies with 3 μm height and 2 μm width.

The ability of the surface to repel bacteria is founded in engineering surface nano topographies. The bacterial attachment to nanoporous topography is reduced by physiochemical forces, including repulsive, electrostatic, and acid-base forces originating from pores (Feng et al., 2015). Hydrophobic surface coatings exhibiting high water contact angle (WCA) and low surface energy give low drag under flow conditions which reduces the strength of adhesion of bacteria to surfaces, thereby preventing microbial contamination (Linklater et al., 2021). The surface protrusions of anti-biofouling surfaces of lotus leaf entrap air bubbles between structures, acting like a hydrophobic surface with incomplete wetting, repel bacteria that encounter the surface as the air layer reduces the surface area for bacterial anchorage. However, the entrapped air is replaced by water or other fluids when immersed in a liquid medium for a prolonged period (Hwang et al., 2018). The wings of dragonflies not only exhibit antibacterial activity but also illustrate anti-adhesive properties (**Figure 2B**). The moderately dense nanoscale features reduce bacterial adhesion due to the reduced contact area between bacteria and the surface; bacteria cannot locate the nanostructures for their anchorage (Linklater et al., 2021). The surface features for antifouling are replicated with inspiration from shark skin, exhibiting low drag and resistance to the adhesion of bacteria. Shark’s surface feature in the form of the diamond pattern was replicated onto polydimethylsiloxane elastomer in Sharklet™ with features of 2µm wide channels, 2µm inter-feature spacing with the height of 3µm and lengths ranging from 4µm to 16µm, incrementing by 4µm (Carman et al., 2006). Anti-adhesive property is also enhanced by the mucous on shark skin, providing lubricating and antifouling benefits (Bixler and Bhushan, 2012). Sharklet™ exerts mechanical stress on the encountering bacteria causing a stress gradient to develop along the bacterial surface (**Figure 3**). The normal cell functions are disrupted under the stress gradient, impelling bacteria to spend energy to adjust the contact area to equalise the stresses. It becomes thermodynamically unfavourable for the bacteria to expend much energy to counteract stress, directing them to search for a different surface to attach (Chung et al., 2007). This creates a natural anti-adhesive surface. *In vitro* and *in vivo* studies with rat models show effective multifold reduction in *S.aureus* and *P.aeruginosa* adherence to micropatterned percutaneous medical device surface (Xu et al., 2017). It also reduced methicillin-sensitive *Staphylococcus aureus* (MSSA) and MRSA contamination of medical devices by 97% and 94%, respectively (Mann et al., 2014).

### 3.2 Antimicrobial touch surfaces

High-touch surfaces are a source of microbial pathogens that often prove to be the origin of HAI. Antimicrobial touch surfaces attempt to reduce microbial contamination on most frequently touched surfaces, primarily of interest in a hospital environment. Copper and its alloys have broad-spectrum antimicrobial activity against bacteria, fungi, and viruses, including SARS-CoV-2, constantly killing 99.9% of pathogens within two hours of contact (Cortes and Zuñiga, 2020). The U.S. Environmental Protection Agency approved copper and its alloys as antimicrobial public health materials widely used in antimicrobial coatings. Copper can inhibit the germination of fungal spores, including *Candida albicans* and hence has been recommended to replace aluminium coils in air conditioners in hospitals to reduce the susceptibility of patients to fungal diseases efficacy of copper antimicrobial touch surfaces in clinical settings has been studied and recommended for use in near-patient environments to decline the risk of transmission (Weaver et al., 2010). The antimicrobial activity of copper is attributed to the release of copper ions upon the chemical decomposition of the material (Villapún et al., 2016). Copper ions destroy microbes by damaging the cell membrane integrity, directly degrade bacterial proteins and induce a Fenton-like reaction which releases hydroxyl ions that interact with DNA, proteins, and enzymes, peroxidise lipids leading to membrane damage. Copper alloys used as anti-microbial touch surfaces reduce horizontal gene transfer (HGT), thus effectively killing the pathogens on the surface and curbing the spread of antimicrobial resistance by HGT (Warnes et al., 2012). Apart from using copper for frequently touched surfaces, copper taps, and pipes can also be fitted in hospitals to reduce water-borne pathogens and associated diseases. Copper Armour™, a novel coating embedded with copper particles, has been developed recently as a self-sanitising coating that complements infection control strategies in healthcare settings (Montero et al., 2019). Silver is also highly recognised for its antimicrobial properties. However, due to the high cost of silver, it is mainly used in the form of nano-formulations and in applications that only require small concentrations.

### 3.3 Self-cleaning and polishing surfaces

**Figure 4:**
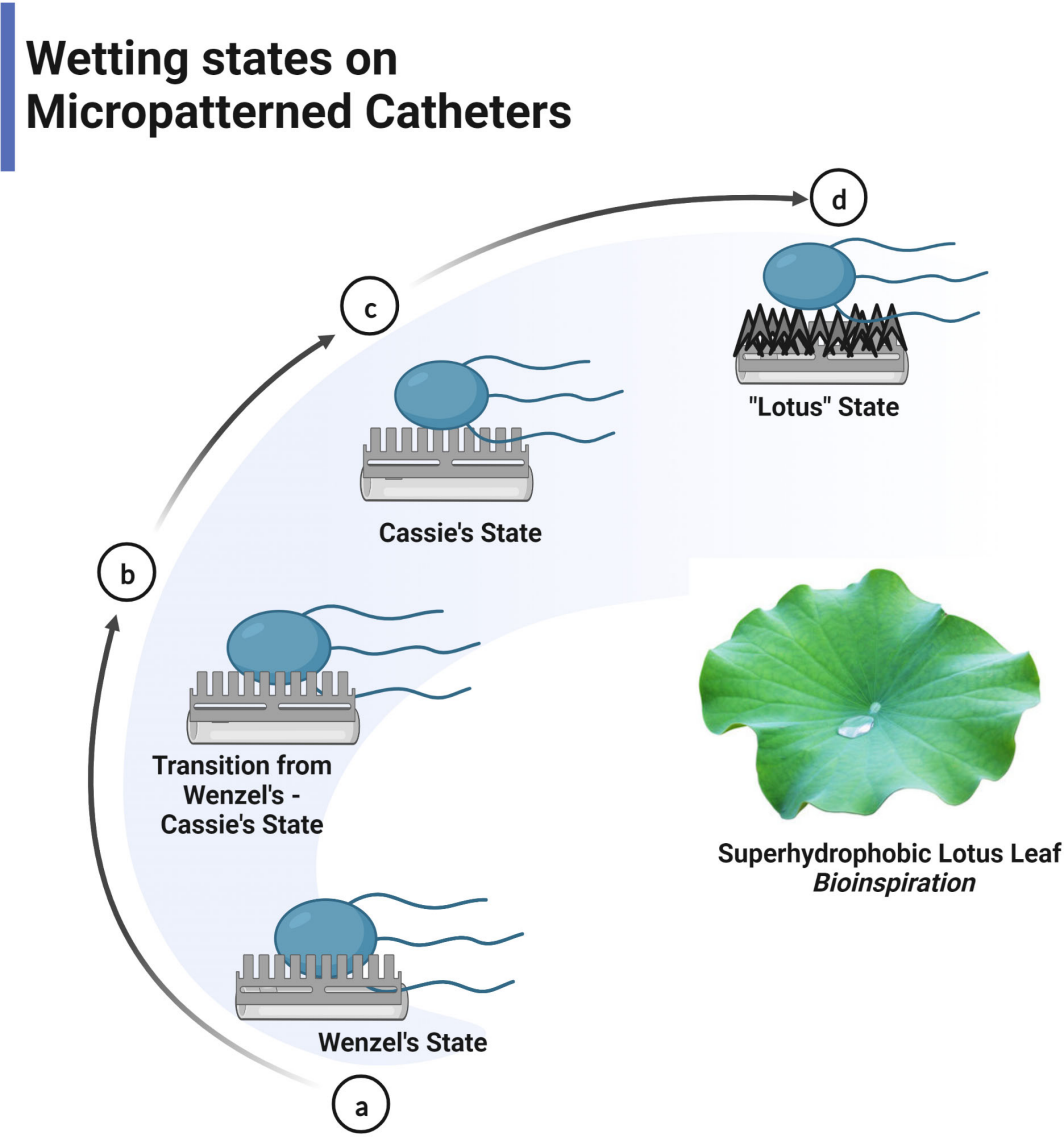
Liquid droplet follows Wenzel or Cassie’s state on the surface. **(A)** Wenzel model- contact angle <150° **(B)** Transition from Wenzel to Cassie’s state **(C)** Cassie’s model > 150° (superhydrophobic state) **(D)** Lotus effect- special Cassie’s state.

The self-cleaning property of the superhydrophobic surface removes biofouling by controlling wettability and particle adhesion confined in surface roughness (Wisdom et al., 2013). Self-cleaning behaviour was extensively exhibited in lotus leaves (Lotus effect), which repels water that rolls off the surface, picking up all the contaminants, including microorganisms leaving behind a clean surface (Wu et al., 2021). The concept of superhydrophobicity revolves around two models, namely the Wenzel model and Cassie–Baxter, where liquid droplet penetratesthe nanopillar in the former model and does not penetrate in the latter (**Figure 4**) (Erbil and Cansoy, 2009). The models are used to optimise the contact angle and surface roughness for obtaining a superhydrophobic surface by assessing the contact area (Parvate et al., 2020). A water droplet on the superhydrophobic lotus leaf exhibits a cassie state contact angle of 164°, low contact angle hysteresis of 3° degrees, and a low tilting angle (TA) of less than 5° for the impending motion of water droplets (Koch et al., 2009). The water-repellent nature of lotus leaves is due to nanoscale epicuticular wax crystalloids on the epidermal papillae rendering microroughness and reduced adhesion of contaminating particles (Barthlott and Neinhuis, 1997). The nanostructures on the micro-papillae with a diameter of ~120nm heighten the surface roughness, reducing the contact area of contaminants and water droplets and endowing low adhesion to the surfaces (Feng et al., 2002). Water droplets balance on the tips of wax crystalloids with air entrapped in the troughs between papillae, increasing the water/air interface and resulting in strong water repulsion (Dettre and Johnson, 1964). The contaminants, including microorganisms on the surface, adhere to the encountering rolling water droplet due to higher adhesion energy and are carried away, leaving a clean surface. The lotus effect has been widely replicated for medical devices to prevent the adhesion of pathogens and biofilm formation. It is supported by the fact that most microorganisms require a wettable surface for adhesion and biofouling (Koch et al., 2009). The lotus-inspired self-cleaning effect imposed on the TiO_2_ nanotubes restricted the surface adherence of *S.aureus and E.coli*, thereby preventing biofilm formation (Patil et al., 2018). It is noted that the superhydrophobic surfaces tend to retain their anti-biofouling property only for a short duration of 4hrs when exposed to pathogens; by the end of 24hrs, the bacterial attachment is about 95-99% due to the loss of air bubble trapped within the intervening space of superhydrophobic structure (Hwang et al., 2018). The major drawback is that the nanostructures causing superhydrophobicity are fragile and easily damaged by mechanical abrasion, leading to reduced WCA and superhydrophobicity. Hence, for applications, high mechanical strength and low-density carbon nanotubes (CNT) with epoxy resin composites were used to fabricate superhydrophobic surfaces possessing low contact angle hysteresis (Jung and Bhushan, 2009). Mechanically robust superhydrophobic surfaces have been realised with simple hierarchical micro-nano structures where nanostructure provides a lotus effect and microscale structures provide durability. The microstructure acts as an interconnected armour harbouring the nanostructures in inverted pyramidal pockets, preventing damage to the nanofeatures by abradants larger than the microstructures, including sandpaper and sharp blade. These surfaces resist shear force and vertical pressure, and regardless of 1000 abrasion cycles, harsh conditions like high temperature (100° C), high velocity of water jet and high humidity exhibit superhydrophobicity with a static WCA of 150° and the TA of fewer than 12° degrees (Ivanova et al., 2012).

The super hydrophobic self-cleaning mechanism is also exhibited by cicada wings possessing waxy coated, hexagonally packed dense nanostructured surface with an average WCA of 158.8° degrees (Watson et al., 2008; Ivanova et al., 2012). The surface of cicada wings was exposed to various gram-negative and gram-positive bacteria and was proved to be highly effective against gram-negative than gram-positive bacteria (Hasan et al., 2013). The sliding water droplet removes the contaminants, similar to the lotus leaf effect. These insects also demonstrate an intriguing autonomous self-cleaning effect by the condensed dew droplets, independent of environmental water supply and control by the gravity. In the presence of water vapour, contaminants are partially or enclosed by the dew condensates. Due to the acquired surface energy, the dew condensates coalesce and jump on the superhydrophobic surface. As a result, the contaminants are spontaneously eliminated from the surface by the self-propelled jumping motion of the dew condensates (Wisdom et al., 2013). In particular, self-cleaning by jumping condensate phenomenon effectively removes adhered bacteria by challenging adhesion involving van der Waals forces.

Rice leaves (*Orysa sativa*), butterfly wings (*Morpho aega, Morpho didius*), and duck feathers (Anatidae) illustrate self-cleaning by superhydrophobic unidirectional wettability with low adhesion properties. This self-cleaning method combines anisotropic flow resulting in low drag from shark skin and a lotus effect (Bixler et al., 2014). The water droplets on the surface easily roll out of the surface along with the rice leaf papillae or radially outward direction but adhere to the surface in the opposite direction. Rice leaves have a transverse sinusoidal arrangement of longitudinal ridges providing anisotropic flow. The longitudinal ridges consist of micropapillary with waxy nanobumps facilitating superhydrophobicity with WCA of 164°, lowest contact angle hysteresis at 3° degrees and low adhesion properties enhancing self-cleaning. Similarly, anisotropic flow is facilitated by shingle-like scales in butterfly wings, and microgrooves on scales provide superhydrophobicity with WCA of 152°, and water droplets roll off the surface at a tilted angle of 9° degrees (Zheng et al., 2007). The porous structure and preening oil coating on the duck feathers furnish a superhydrophobic character. The porous structure is established by the branches of feathers further dividing into barbules, enhancing the air-water interface and resulting in water repellence (Cassie and Baxter, 1944). Rice leaf replicas were fabricated by a commercial hot embossing technique using micropillars and ribs on urethane polymer with a WCA of 155° showed 95% contaminants removal in water droplet wash experiments (Bixler et al., 2014).

The rear side of the fish scales and shark skin also exhibit self-cleaning effects potentiated by hydrophilicity and oleophobicity. These surfaces prevent microbial adhesion and biofouling through complete water wettability and enhanced oil repellence, enabling water to get in between contaminant and surface, washing away the impurities. Fish scales exhibited hydrophilicity and super oleophobicity (oil contact angle of 156°) stemming from the micro-nano hierarchical structures and were replicated on silicon wafers by lithography technique (Liu et al., 2009). The micro-nano hierarchical structures entrap water, preventing contaminants from contacting the surface. Likewise, the super hydrophilicity and superoleophobicity properties of lotus leaves are contributed by convex micropapillary covered with nano grooves in the range of 200-500 nm (Cheng et al., 2011). Sharkskin possesses dermal denticles containing parallel riblets along the swimming direction, facilitating a typical self-cleaning through hydrophilicity and anisotropic fluid flow, leading to low drag (Yu et al., 2020). As the water flows, vortices develop on the surface, causing high shear stress lifted by the riblets, exposed to only the tips of riblets. The minimised shear stress reduces drag across the surface, enabling swift movement of water adjacent to the shark skin and washing away the adhered microorganisms. The Sharklet AF™ bioengineered based on shark skin’s microtopography is evidenced to be effective in preventing colonisation and biofilm formation (Chung et al., 2007). The Riblet patterns were also studied for drag reduction efficiency on various materials (Bixler and Bhushan, 2012).

**Figure 5:**
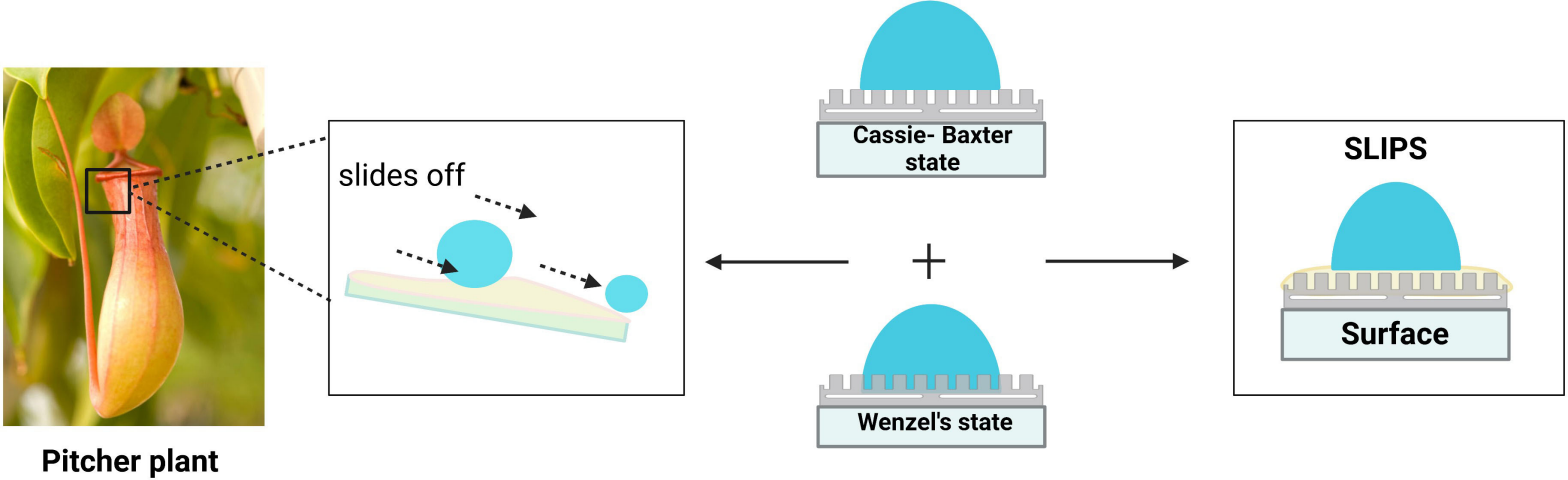
*Nepenthes* pitcher plant-inspired Omniphobic surface.

**Figure 6:**
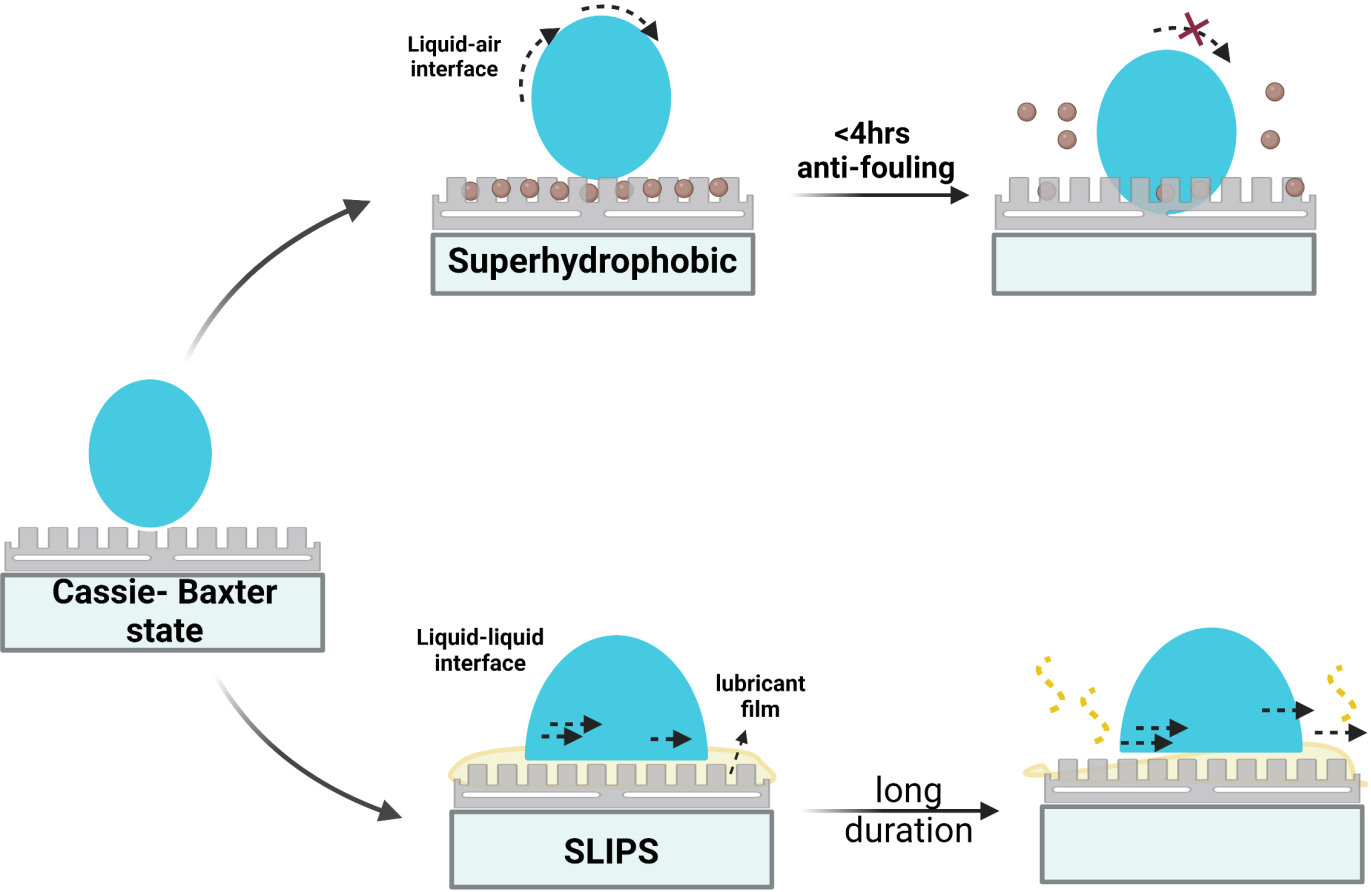
Comparison of Slippery liquid-infused porous surface (SLIPS) to superhydrophobic surface.

Omniphobic surfaces, named slippery liquid-infused porous surfaces (SLIPS) inspired by *Nepenthes* pitcher plants, are similar to superhydrophobic surfaces, wherein an additional component is a lubricating film on the surface (**Figure 5**). The surface displays self-cleaning by repelling various simple, complex, broad-range surface tension liquids like water, crude oil, and blood. In SLIPS, the rough substrates in the micro-nano scale immobilise thoroughly wetting and incompressible lubricating fluid resulting in a homogeneous, molecularly smooth surface with exceptional low friction that repels impacting immiscible liquids. The presence of lubricating fluid in SLIPS counteracts the downside of superhydrophobic surfaces like poor stability, low mechanical strength, and durability due to loss of entrapped air over a short period of time, leading to the exposure of rough surface favouring bacterial attachment is overcome by the presence of lubricating fluid in SLIPS (**Figure 6**) (Wang and Guo, 2020). The combination of substrate and lubricating film must be worked out based on interfacial energies and physical and chemical properties. Pitcher plant-inspired synthetic liquid-repellent surface was developed with ordered poly-fluoroalkyl silane functionalised nano-post array and random teflon based porous nanofiber network with perfluorinated liquids (e.g. Fluorinert FC-70) as lubricating film. They exhibited low CAH of less than 2.5° and low TA (< 5°) for various repelled liquid droplets. SLIPS show impressive pressure stability and self-healing upon recurring, large-area damage by abrasion or impact within 1 second (Wong et al., 2011). SLIPS were also applied for the enamel surface, and results revealed significant inhibition of salivary mucins adsorption, adherence of *Streptococcus mutans in vitro*, and dental plaque formation *in vivo *(Yin et al., 2016). Owing to the repellence of blood and other liquids on the surface, omniphobic coating has been applied to tubing and catheters. A flexible molecular layer of perfluorocarbon is covalently tethered to the device surface and further infiltrated by a mobile film of medical-grade perfluorodecalin to produce an omniphobic coating with a TA of only 0.6 degrees. This coating effectively prevents the adhesion of fibrin, platelets, and their activation and also reduces the adhesion of *P. aeruginosa* and *E. coli* bacteria and subsequent biofilm formation by eight folds over 6.5 weeks, thereby preventing thrombosis and biofouling *in vitro* and in *in vivo* pig model (Leslie et al., 2014). The impressive characteristics of omniphobic surfaces can be compromised gradually owing to lubricant evaporation and shear stress under high flow conditions. Hence, a self-replenishing SLIPS with an integrated lubricant reservoir called nanotubes (combination of nanohole and nanopillar) was fabricated using non-volatile and high-viscous lubricants to enable prolonged operation (Wong et al., 2011; Laney et al., 2021).

#### 3.3.1 Photocatalytic coatings

Self-cleaning surfaces have been realised with photocatalysts like TiO_2_, ZnO, and CdS coated on medical devices and equipment to achieve antimicrobial surfaces in near-patient environments and highly contaminated areas in hospitals. TiO_2_ is considered a promising application as a super hydrophilic photocatalytic coating due to non-toxicity, environmental friendliness, chemical inertness in the absence of light, photostability, durability, abundance, and low-cost production. TiO_2_ semiconductor, upon irradiation with UV light, decomposes the organic contaminants adsorbed on the surface by OH^-^, H_2_O_2_, and O_2_^-^ ROS generated from photocatalytic oxidation activity. Subsequently, the decomposed contaminants are washed away from the surface and sterilised by sheeting water owing to super hydrophilicity induced by photons. TiO_2_ demonstrates a broad-spectrum bactericidal effect and kills yeast and green algae (Padmanabhan and John, 2020). Moreover, photocatalysis of TiO_2_ brings down air pollutants like nitrogen oxides and boosts air quality like plants (Nishimoto and Bhushan, 2013). *S.aureus* and *Pseudomonas putida*, established on flat and porous glass functionalised with TiO_2,_ were killed with 99.9% efficiency owing to membrane damage within 2 hours of irradiation in the range 290-400 nm (Jalvo et al., 2017). Phosphorous and fluorine-modified TiO_2_ coating revealed photocatalytic activity against *E. coli*, *S.epidermidis*, and *Pseudomonas fluorescens* with 99% efficiency within 10 minutes under UV-A illumination (Yan et al., 2020). Medical devices can be coated with titania nanosheet with a surface roughness of 0.95 nm, as improved contaminant removal by photocatalytic oxidation activity and photoinduced super hydrophilicity were observed. Similarly, increased photocatalytic oxidation activity was observed with ordered titania nanotubes and graphene/TiO_2_ hybrid films. In general, the heightened photocatalytic effect of TiO_2_ can be realised in the form of nanocrystalline particles, nanowires, nanotubes, and nanoflowers with dimensions in the range of 1-100 nm, due to effective oxidation and reduction processes releasing ROS in large amounts (Ragesh et al., 2014). It was reported that a thin layer of WO_3_ deposited on TiO_2_ coating upgrades sensitivity to weak UV light intensity for the photoinduced super hydrophilic conversion (Nishimoto and Bhushan, 2013).

The current research trend focuses on tuning the excitation wavelength for the photocatalytic activity to the visible region by doping with metals and non-metals, hybridisation with organic and inorganic groups, and using the dye photosensitisation method. N-doped TiO_2_ films impregnated with synergistic silver nanoparticles, under white light presented, antimicrobial photoactivity against gram-positive and gram-negative bacteria, particularly MRSA and *E.coli* (Dunnill et al., 2011). TiO_2_ doped with Bi and N, coated on dental implants, demonstrated photocatalytic anti-bacterial properties upon visible light excitation and was retained even in darkness. It showed bacterial reduction and cleared biofilm formed by *Streptococcus sanguinis* and *Actinomyces naeslundii* (Padmanabhan and John, 2020). Copper (0.5 mol%) doped TiO_2_ proved effective against *E.coli* and *S. aureus* with 5 fold reduction in bacterial viability within 30 mins when excited with visible light (Mathew et al., 2018). TiO_2_ co-doped with fluorine and copper demonstrated antibacterial activity against *S. aureus* following excitation with visible light-inducing photocatalysis combined with copper ion toxicity. Fluorine dopant renders sensitivity to visible light for photocatalytic activity, and co-doping with copper dramatically improves the efficiency of bacterial inactivation in both light and dark conditions due to the intrinsic antimicrobial activity of copper ions, acting in synergy with the photoactivity of fluorine-doped TiO_2_ (Leyland et al., 2016). Thus, the difference in the efficiency of photocatalytic activity against gram-positive and gram-negative bacteria owing to variations in cell wall composition, gram-negative bacteria being more resistant to TiO_2_ photoinduced bactericidal activity, can be mitigated with the introduction of synergistic antimicrobial metal ions like copper, silver, gold into the coating. Photocatalytic coatings can also be incorporated into filter systems of water purifiers to eliminate pathogens in water.

#### 3.3.2 Self-polishing coatings

The biofouling of ship hulls is prevented by conventional self-polishing coatings on surfaces, releasing toxic biocides like tributyltin (TBT) and cuprous oxide on the gradual erosion of the coating. The coating is degraded over 10-100nm thick due to hydrolysis by seawater, and the bloated coating gets ablated by water, releasing biocides in the vicinity and eliminating the biofouling from the ship’s surface. The constant surface erosion results in the exposure of fresh biocides and self-renewal of a clean surface. However, the potential side effects of this coating include the development of resistant microbes, marine pollution, and sexual pattern change in marine organisms as consequences of the unnecessary release of biocides. The coating has to be renewed periodically. Tributyltin and other toxic coatings are also banned and restricted by International Marine Organisation (IMO) because of their toxic effects (Bieser et al., 2011). Consequently, much research effort has been devoted to promising self-polishing coating with natural antifoulants. The self-polishing coatings in the marine field can be extended to the medical field, where parallel toxicity problems and the emergence of antimicrobial resistance persist, complicating treatments and prevention of device-related and hospital-acquired infections. Recently, the potential of natural compounds has been explored to address AMR owing to its minimal side effects, synergistic activity with existing antimicrobials, sensitising resistant bacteria to antimicrobials, and reversing the AMR (Álvarez-Martínez et al., 2020). These natural antifouling compounds can be loaded into natural biodegradable resins like water-soluble resin, which has the potential for extended-release. Polycaprolactone (PCL)–Polyurethane (PU) copolymer rosin blend incorporated with butenolide presented an antifouling self-polishing effect for up to 3 months. The release of butanolide due to the hydrolysis of ester linkages and self-renewal of the surface contributes to the self-polishing of the surface (Ma et al., 2017). Borneol extracted from medicinal herbs like chamomile, and lavender synthesises isobornyl methacrylate (IBOMA) polymer with broad-spectrum antibacterial activity apart from anti-inflammatory, anti-thrombogenic and vasorelaxant effects. Self-polishing coatings can be produced with IBOMA polymer incorporated with antifouling agents. On slow degradation, release borneol and antifouling agent, thereby self-renewing the surface and preventing bacterial adhesion (Hu et al., 2020). These coatings can find applications in the short-term usage of urinary catheters. Surfaces heavily contaminated with microorganisms can be refreshed by detaching the outermost contaminated layer. Such self-decontamination surfaces are achieved with layer-by-layer deposition of alternating dextran aldehyde and carboxymethyl chitosan connected with imine linkages, which are cleaved in response to acidic conditions stimulated by bacterial biofilms (Xu et al., 2018). A self-polishing coating based on cellulose polymer has been produced, which erodes in response to cellulase produced by various microbial strains. Thus, the release of antifoulants is regulated by the adherence of microorganisms (Bieser et al., 2011). Such self-polishing coatings are promising for mitigating bacterial adherence and biofilm formation within a few hours or days after implantation. They are effective for coatings on implants purposed to integrate with host tissues like orthopaedic implants, temporary implants and devices, walls, bed rails and near patient highly touched surfaces.

## 4 Conclusion

The device-related healthcare-associated infections plague the medical field, and bacterial contaminations are inevitable despite following aseptic conditions while performing the procedures. The current strategies of local or systemic administration of antibiotics are associated with extreme cytotoxic effects on the patients. The various release-based antimicrobial coatings for devices also suffer from limitations, including burst release of antimicrobial compounds, precocious degradation within the body, and decreasing antimicrobial efficacy due to elution of antimicrobial agents resulting in susceptibility to infections. Inappropriate usage of antimicrobial agents induced the emergence of antimicrobial resistance, posing a world of challenges to researchers and engineers to be solved for the realisation of next-generation devices. Multifunctional approaches inspired by nature provide convincing solutions to these challenges, and various concepts of antibacterial surfaces, as discussed in this review, are validated against a few leading pathogens. However, an ideal antibacterial approach does not exist, and direct implementation of natural design parameters for all practical applications is impossible. This requires optimisation for various applications involving different surface materials and working conditions. The dimensional parameters and aspect ratio of micro-nano topographical structures of antibacterial surfaces and anti-adhesive surfaces must be determined for bactericidal effect against different sized bacteria apart from broad-spectrum antibacterial effect. Incorporating them in multifunctional surfaces combining anti-adhesive and killing strategies could meet clinical demands and abate HAIs. Nanostructured antibacterial surfaces integrated with self-cleaning properties can effectively clean off the debris of killed bacteria, indefinitely sustaining the functionality and efficiency of the surface. The surface features reviewed in this article are fragile and can be damaged under mechanical stress. Hence, hierarchical mechanically robust designs that have been reported must be considered while modelling medical devices for applications. The antibacterial surfaces can also be fabricated with adhesive back for easy implementation on existing devices. New high-throughput technologies and data, including omics, computational modelling, and network pharmacology, can be employed to identify the synergistic activity between natural compounds and the resulting systemic effects for developing promising combinations for incorporation in self-polishing surfaces. Further, the prolonged controlled release of natural antifoulants and the rate of the detachment of the outermost layer of the self-polishing surface are essential issues to be considered. In the future, multifunctional surfaces combining various modification concepts can be engineered to overcome the limitations of other approaches and effectively mitigate infections. Also, the long-term efficacy and durability of these next-generation surfaces must be determined and haven’t been reported in the literature. The developments in these antibacterial surfaces over the past decade have spurred further investigations and would aid in combating antimicrobial resistance and healthcare-associated infections.

## Author contributions

Original draft manuscript preparation and writing: SR and HS; Image editing: SR, HD, and AS; Reviewing and editing: KS, RD, and APS. All authors contributed to the article and approved the submitted version.
